# Geostatistical linkage of national demographic and health survey data: a case study of Tanzania

**DOI:** 10.1186/s12963-021-00273-0

**Published:** 2021-10-28

**Authors:** Eun-hye Yoo, Tia Palermo, Stephen Maluka

**Affiliations:** 1 Department of Geography, State University of New York at Buffalo, Buffalo, NY USA; 2 Department of Epidemiology and Environmental Health, State University of New York at Buffalo, Buffalo, NY USA; 3 College of Education, University of Dar es Salaam, Dar es Salaam, Tanzania

**Keywords:** Geostatistical linkage methods, Demographic and Health Survey (DHS), Service Provision Assessment (SPA), Misclassification error

## Abstract

**Background:**

When Service Provision Assessment (SPA) surveys on primary health service delivery are combined with the nationally representative household survey—Demographic and Health Survey (DHS), they can provide key information on the access, utilization, and equity of health service availability in low- and middle-income countries. However, existing linkage methods have been established only at aggregate levels due to known limitations of the survey datasets.

**Methods:**

For the linkage of two data sets at a disaggregated level, we developed a geostatistical approach where SPA limitations are explicitly accounted for by identifying the sites where health facilities might be present but not included in SPA surveys. Using the knowledge gained from SPA surveys related to the contextual information around facilities and their spatial structure, we made an inference on the service environment of unsampled health facilities. The geostatistical linkage results on the availability of health service were validated using two criteria—prediction accuracy and classification error. We also assessed the effect of displacement of DHS clusters on the linkage results using simulation.

**Results:**

The performance evaluation of the geostatistical linkage method, demonstrated using information on the general service readiness of sampled health facilities in Tanzania, showed that the proposed methods exceeded the performance of the existing methods in terms of both prediction accuracy and classification error. We also found that the geostatistical linkage methods are more robust than existing methods with respect to the displacement of DHS clusters.

**Conclusions:**

The proposed geospatial approach minimizes the methodological issues and has potential to be used in various public health research applications where facility and population-based data need to be combined at fine spatial scale.

## Background

As a part of the Demographic and Health Surveys (DHS) project, Service Provision Assessments (SPA) provide a comprehensive overview of health service delivery in low- and middle-income countries (LMICs). When these national level surveys of health facilities from SPA are combined with individual- or household-level surveys on population, fertility, family planning, reproductive health, child health, and nutrition [19], they can provide critical information to address key programmatic and targeting questions related to population health in LMICs [13].

Several studies have proposed analytical methods to establish a link between survey respondents in household data (clusters from DHS) and individual facilities from SPA based on the Global Positioning System (GPS)-referenced location information of each dataset. For example, Hong et al. [11] used a nearest facility linkage method, Wang et al. [31, 32] and Wang et al. [33, 34] conducted a buffer analysis for service-environment, while others [28, 30] used a combination of administrative boundary link, Euclidean buffer link, road network link, and kernel density estimates. These studies, in common, aimed to address key programmatic and policy questions about program impact and targeting related to population health, such as newborn health, vaccination, contraceptive use and access, adolescent sexual and reproductive health by combining these two datasets [2]. DHS has also been linked with additional sources of health facility data other than SPA in previous studies [15, 17, 23, 29] using geospatial linkage methods to examine outcomes including place of delivery, antenatal care, contraceptive use, neonatal mortality, and newborn care.

However, existing methods were recommended for analyses at regional levels [2, 18, 28] due to known limitations of the survey datasets, such as (1) the SPA facility survey includes only a subset of all health facilities in a country and (2) DHS clusters are intentionally displaced in space for privacy protection. Typically, SPA is conducted as a nationally representative sample of facilities whose design allows calculation of sub-national estimates of indicators by sectors, facility level, and administrative unit [3]. Only in a small number of countries has a facility census approach been used [32]. As documented elsewhere [2, 11, 28], substantial misclassification likely occurs if the SPA are directly linked to DHS at the cluster level. For example, the linked data may underestimate the availability of health facilities (or access to health services) and misclassify the nearest facility from household clusters [28].

To address the incompleteness of SPA in their linkage to DHS surveys at disaggregated levels, one can consider a geospatial approach based on spatial interpolation. Spatial interpolation has long been used in geographic information science (GIScience) to estimate the magnitude of the features at locations without data using known values at a number of selected locations [16, 26]. Among many interpolation methods, the geostatistical technique, also known as Kriging, is considered optimal. It supplies the best linear unbiased estimates, while exploiting spatial dependence in the phenomenon of interest [5, 6, 12].

Specifically, spatial dependence in Kriging embodies two types of effects [14]. The first-order effects, known as a trend or drift, quantify the large-scale spatial variation of the data over the study area. For example, high-quality health services are more likely available at hospitals than other types of health facilities. In LMICs, hospitals are typically located where a set of geographic, socio-economic, and demographic conditions are optimal [1, 8]. Thus, we expect that a trend of health service availability (or quality) can be modeled based upon location-specific environmental characteristics. The second-order effects capture localized spatial structure in the data, including clusters of similar values or spatial autocorrelation [10]. The spatial autocorrelation in the data, such as the similarity of health service availability of adjacent SPA facilities, increases the predictive accuracy of Kriging [4] and potentially allows overcoming the limitations of accessing a sample instead of a census of health facilities. Kriging captures both the first-order effect and the second-order effect of the spatial variation of health services availability.

The effect of geographic displacement of DHS cluster data on their linkage of health facilities in SPA has been assessed primarily focusing on geospatial proximity, which frequently imposes over-simplified assumptions. For example, the administrative boundary method links the two data sets assuming that the boundary dominates a choice of health facility. Except for in a few counties where the health care system was built upon administrative boundaries, however, administrative boundaries are delineated for a purpose of governing and not for delivering efficient health services. Similarly, kernel density or road networks are purely based only on the location of health facilities regardless of the physical and social/political environments surrounding DHS clusters, such as the presence of water bodies or mountains. Further investigation of the effect of geographic displacement of DHS clusters is needed.

In the present study, we developed a novel geostatistical approach to link health facility survey data with geocoded DHS clusters at a disaggregated level (i.e., the cluster-level). The proposed approach accounts for both the geographic configuration of DHS clusters and SPA facilities, as well as the local environmental conditions of the sampled health facilities in SPAs. We demonstrated the effectiveness of the proposed approach by estimating the general service readiness (SR) of each health facility [35] as an example and predicting the SR availability in any given DHS cluster. We also assessed the effect of geographic displacement of DHS cluster data using spatial simulation [37]. To assess the predictive model performance, we used a unique data set of the census of health facilities in the two regions in Tanzania.

## Methods

### Study setting

**Fig. 1:**
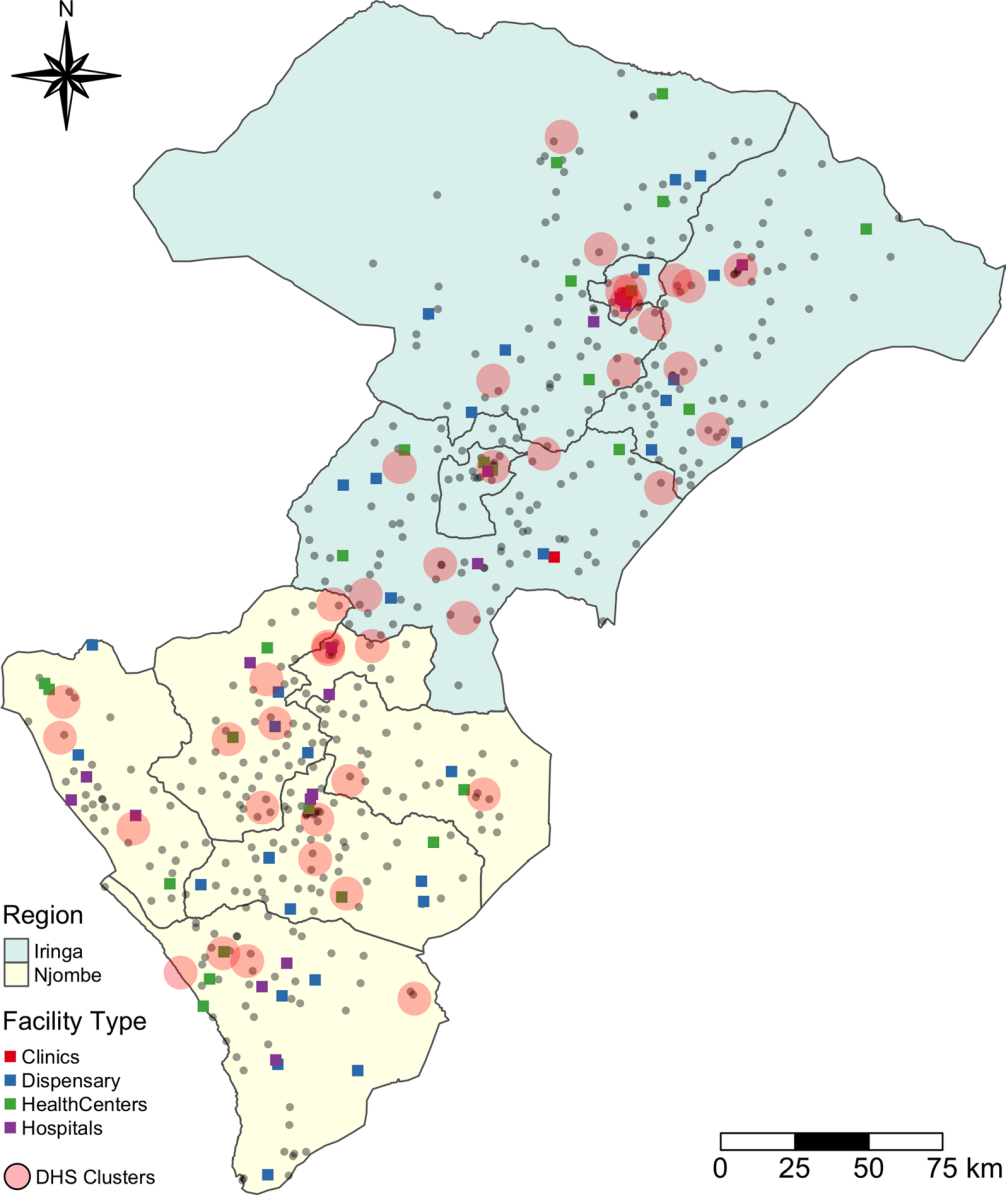
The census of health facility overlaid with SPA sampled facility within the administrative boundaries of Iringa and Njombe (shaded with green and yellow color, respectively). The gray line denotes the boundary of districts (administrative level 2) within each region. The DHS clusters are denoted by red circles

There are 20 regions in mainland Tanzania and we focused on the two regions: Iringa and Njombe. These were selected because two of the authors have extensive contextual knowledge of these regions. The two regions, located in the Southern Highlands in the southwestern part of the country, are comprised of multiple districts and town councils as shown in Fig. 1.

In mainland Tanzania, the public health care system is organized as follows. Primary health care services are the most common and comprise community-based health activities (disease control programs, community outreach, etc.), village health services (often health services offered in home by village health workers), dispensaries and health centers. Dispensaries are generally run by a clinical assistant and can provide preventive and curative services, while health centers can admit patients and provide some surgical services planning [20]. For example, dispensaries provide maternal and child health care, treat simple medical problems during pregnancy such as anemia, assist with normal deliveries, and offer basic outpatient curative care to between 6,000 and 10,000 people. Health centers are normally run by clinical offers and serve population of approximately 50,000 people. Health centers are intended to provide preventive care, but also often have between 10 and 20 beds and offer reproductive health services and minor surgery. Clinics are another category of health facilities at the primary level which are privately owned and offer general and specialist examinations and outpatient treatment services. At the secondary level, council hospitals provide health care and medical and basic surgical services, while regional hospitals provide specialist medical care. Finally, at the tertiary level, zonal and national hospitals provide advanced medical care and training in medical, paramedical, and nursing care. The two entities jointly responsible for the delivery of public health services are the Ministry of Health and Social Welfare and the Prime Minister’s Office.

### Data sources

Data used in the present study came from multiple and publicly available sources. The 2014–2015 Tanzania SPA Provision Assessment (TSPA) provides information on availability of basic and essential health care services and readiness to provide quality services [20]. The TSPA is a sample of formal-sector health facilities in Tanzania. The sampling frame for the survey is a master list of 7102 facilities in Tanzania and Zanzibar, including hospitals, health centers, clinics, and dispensaries. These include government, private for-profit, parastatal, and faith-based facilities. A total of 1200 facilities were selected for the survey to represent a nationally representative sample by facility type and managing authority. Hospitals were oversampled as they exist in comparatively small numbers. In each facility, data were collected using a facility inventory questionnaire, health provider interview, observation protocols, and exit interviews for antenatal care and family planning clients. Fieldwork was carried out between October 2014 and February 2015.

For the 2015–2016 Tanzania Demographic and Health Survey and Malaria Indicator Survey (TDHS-MIS), National Bureau of Statistics (NBS) carried out and supervised fieldwork activities, while ICF International provided technical assistance. Sampling was carried out in two stages. In the first stage, 608 clusters corresponding to enumeration areas delineated for the 2012 census were selected. For the second stage, a complete listing of households in all 608 selected clusters was carried out, and then 22 households were randomly selected from each cluster, yielding a representative probability sample of 13,376 [22]. GPS data were collected at the cluster level, accurate to less than 15 m, although their latitude/longitude positions were randomly displaced to ensure respondent confidentiality. The range of displacement in rural clusters is 0 to 5 km, and then 1% of clusters are displaced with a range of 0 to 10 km. Data collection was carried out by 16 field teams across Tanzania between August 2015 and February 2016.

Census data on all health facilities in Tanzania were obtained from the Ministry of Health, Community Development, Gender, Elderly and Children (MoHCDEC) website (https://www.moh.go.tz/hfrportal). These data include facility name, type, ownership, and GPS coordinates. Data were obtained from the website in 2019 as an up-to-date census of existing facilities. Census data were unavailable for the same year as the TSPA was sampled, although the yearly rate of change in health facility placement is usually very low.

We sourced the gridded population data for Iringa and Njombe regions at 100 m spatial resolution projected for years of 2014 and 2015 from WorldPop database (www.worldpop.org). The road network data were obtained from Humanitarian Data Exchange (https://data.humdata.org/). We classified this dataset originally derived from OpenStreetMap into one of the following classes: primary, secondary tertiary roads, and tracks. We collected the elevation information from Shuttle Radar Topography Mission (SRTM) at the resolution of 3-arc sec, which was produced by National Aeronautics and Space Administration (NASA). We downloaded the digital elevation model (DEM) dataset from Jet Propulsion Laboratory (https://www2.jpl.nasa.gov/srtm/). The land use product was obtained from AFRICOVER project (Di Gregorio & Latham, 2009), which we downloaded from Food and Agriculture Organization of the United Nations (http://www.fao.org/geonetwork/srv/en/main.home). The land cover data originally derived from Landsat satellite images were regrouped into ten types of land uses: built area, woodland, forest, tree savannah, shrub land, sparse herbaceous vegetation, herbaceous crops, shrub crop, flooded vegetation, and water bodies.

**Fig. 2:**
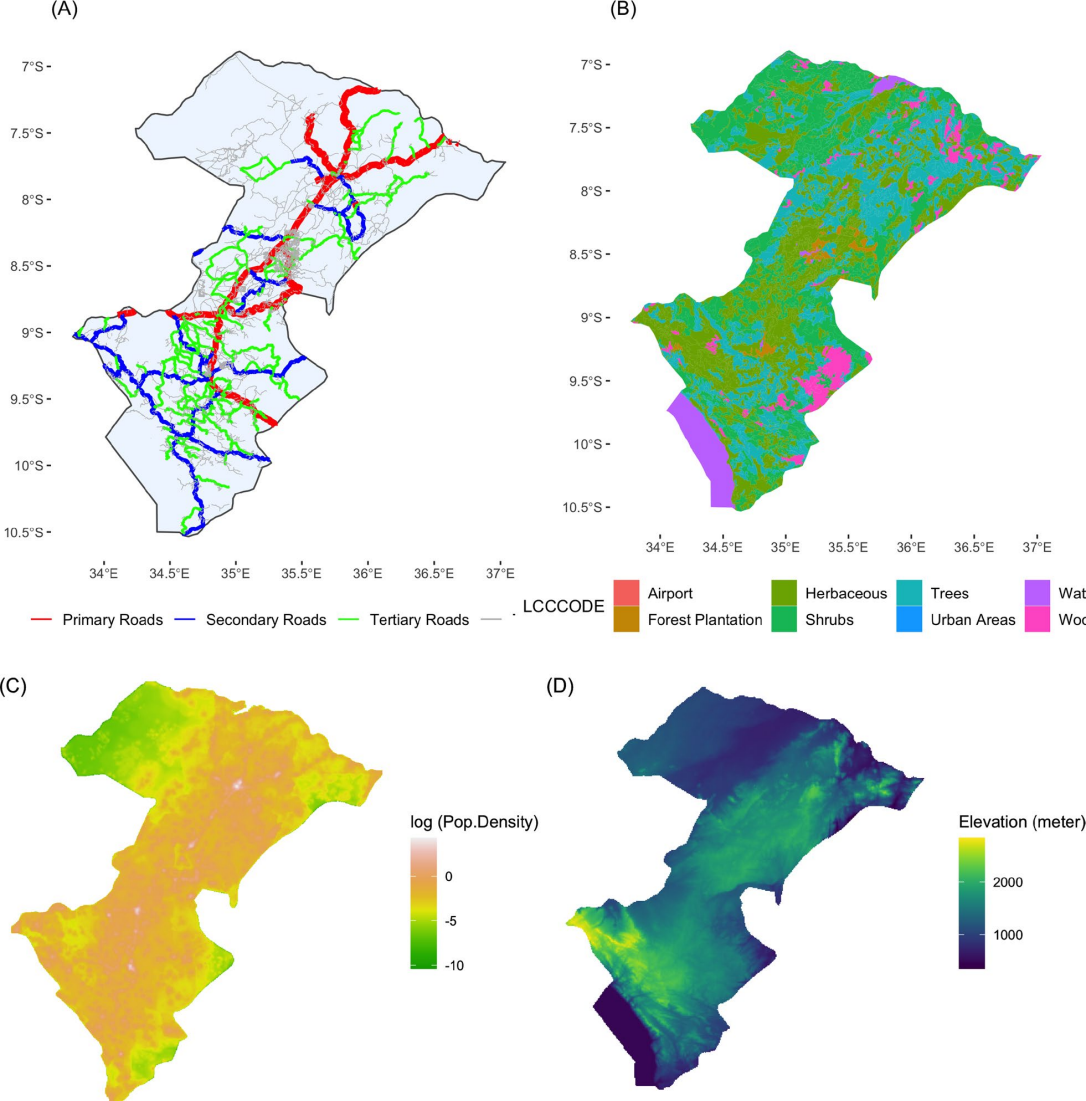
(**A**) Road networks, (**B**) Land uses, (**C**) Population density, and (**D**) Elevation

We processed the raw data for modeling, including the elevation data being resampled at the spatial resolution of 500 m via a bilinear interpolation in Fig. 2D, and other datasets, such as road network in Fig. 2A, land cover information in Fig. 2B, and population density in Fig. 2C.

### SPA data analysis of service readiness

To estimate service readiness, we were guided by WHO standards outlined in the Service Availability and Readiness Assessment Reference Manual [35]. For each facility, we used data from SPA to create a score summarizing service readiness comprising various items related to its operational capacity (see Appendix A for details). If the facility had the specified item, we coded the item as 1 and 0 otherwise. Then for each dimension (amenities, basic equipment, infection prevention, diagnostic capacity, and essential medicines), we created a sub-score ranging from 0 to 1 indicating the average items the facility had for that dimension. Then we created the general service readiness (SR) score by averaging the sub-scores by facility and then multiplying by 100. Thus, the SR score has a potential range from 0 to 100.

### Geostatistical linkage methods and performance evaluation

We aimed to account for both the location-specific conditions, such as elevation, land use types, and population density where the facility is located, as well as the spatial correlation among the general SR scores of health facilities. We assume that health facilities in the study area share common geographic, socio-economic, and demographic characteristics that attain spatial efficiency in the spatial distribution of health facilities [24], given that the cost of developing new health facilities in LMICs is high. For example, both Mishra et al. [21] and Pu et al. [25] identified key factors that are important to consider for optimal locations for new health facilities in LMICs. They include the distance to the nearest health facility, population ratio, connection to road networks, land use type, and elevation.

We expected that the key determinants of health facility sites used in the present study, which were not necessarily exhaustive, would be shared by health facilities that were both included and not included in the SPA survey. Here we addressed the challenge of making a link based only on a sample data in SPA instead of a census of health facilities by borrowing information from the SPA sampled health facilities and their surrounding environmental characteristics. We would also account for both the geographic proximity to health care facilities from prediction locations, including DHS clusters, and the distance between any two SPA facilities. To achieve this goal, we used two kriging algorithms: simple kriging with varying local mean [9] and kriging with an external drift (also referred to as universal kriging) [4, 7, 27, 36]. Both algorithms can represent the spatial variation of the general service readiness via a stochastic surface. The general SR of each facility was considered a regionalized variable whose mean varied in space and was modeled by a local trend, while the remaining variation not captured by the local trend followed a second-order stationarity [12]. Variogram (or covariance) characterizes the second-order stationarity of the regionalized variable where the geographic proximity to health facilities can be realistically modeled. Hereafter, we use the following notation to explain the two kriging models: $\mathbf{u}$ denotes a location in the study area $\mathbf{u} \in A$. The response variable, general SR, is denoted as $Z(\mathbf{u} )$, which is viewed as a regionalized variable and the observed general SR scores at the 77 SPA facilities $[z(\mathbf{u} _i), i= 1, \ldots , 77]$ correspond to a realization of the regionalized variable.

*Generalized linear models for trend estimation*: We estimated the local trend of the general SR using a multivariate linear regression with covariates that summarize the physical environment, socio-economic conditions, and demographic characteristics surrounding each health facility. Specifically, the local trend was modeled as

1 $$\begin{aligned} m(\mathbf{u} )= \beta _0 + \beta _k \sum _{k=1}^4 x_k(\mathbf{u} ) \end{aligned}$$

where the four covariates consist of elevation $x_1(\mathbf{u} )$, log-transformed population density $x_2(\mathbf{u} )$, road density $x_3(\mathbf{u} )$, and the distance from the location $\mathbf{u}$ to the nearest major road $x_4(\mathbf{u} )$. These covariates were selected based on an exploratory analysis where land use derived variables, such as the proportions of land use types per 500 m, were found to be insignificant in explaining the variation of the general SR scores of SPA facilities. We have used the four variables for both the model fit and the prediction: a linear model was fitted at SPA facility sites to estimate the local trend and prediction was made over a grid with a cell size 500 m imposed on the study area. We expect that the local trend model in Eq. (1) predict high general SR score at locations when a similar set of physical conditions are met, and thus overcome the challenges of using a subset of health facility data.

*Estimating spatial structure via variogram analysis*: The variogram analysis was performed using the residuals of the general SR scores obtained at SPA health facility sites. The residual values were computed by subtracting the trend estimate from the general SR scores as $r(\mathbf{u} _i) = z(\mathbf{u} _i) - {\hat{m}}_{SK}(\mathbf{u} _i), i=1, \ldots , 77$. In variogram, the spatial variation of general SR scores depends only on the relative locations of health facilities. That is, the similarities of general SR scores of two health facilities vary as a function of their distance regardless their absolute locations: if a pair of health facilities are away from each other, their SR scores likely to differ, but their SR scores are similar if they are close together.

*Spatial prediction of general service readiness*: Under the model decision of second-order stationarity, the mean of the general SR does not depend on the location and it represents global information shared to all unsampled locations. In the simple kriging with varying local means (SKLM), we replaced the mean *m* by known varying means ${\hat{m}}_{SK}(\mathbf{u} )$ to account for the set of covariates available at each location, which led to the following form of the simple kriging with varying local mean estimator ${{\hat{Z}}}_{SKlm}(\mathbf{u} )$:

2 $$\begin{aligned} {\hat{Z}}_{SKlm}(\mathbf{u} ) - {\hat{m}}_{SK}(\mathbf{u} ) = \sum _{i=1}^{77} \lambda _i^{SK}(\mathbf{u} ) [ Z(\mathbf{u} _i) - {\hat{m}}_{SK}(\mathbf{u} _i)] \end{aligned}$$

where $\lambda _i^{SK}(\mathbf{u} )$ denotes the kriging weight assigned to the general SR score at the *i*-th health facility in the SPA survey. The varying means ${\hat{m}}_{SK}(\mathbf{u} )$ was estimated using a generalized linear models with a Gaussian distribution assumption in the present work. The residual values at locations (grid cells imposed on the study region) were estimated using the simple kriging with varying local means estimator with the residual data $r(\mathbf{u} _i), i = 1, \ldots , 77$. The final estimate of general SR scores were obtained by adding the trend estimate ${\hat{m}}_{SK}(\mathbf{u} )$ to the SK estimates of the residual ${\hat{r}}(\mathbf{u} )$.

Kriging with an external drift (KED) is similar to kriging with the simple kriging with varying local means in Eq. (2) in that the trend is modeled as a linear function of a smoothly varying auxiliary variables, but it is different because the mean $m(\mathbf{u} )$ is not estimated through a regression process prior to the kriging of *Z*. The KED estimator is

3 $$\begin{aligned} {\hat{Z}}_{KED}(\mathbf{u} ) = \sum _{i=1}^{77} \lambda _i^{KED}(\mathbf{u} ) Z(\mathbf{u} _i). \end{aligned}$$

The kriging weights $\lambda _i^{KED}(\mathbf{u} )$ are the solution of the following system of (77+5) linear equations

4 $$\begin{aligned} \sum _{j=1}^{77} \lambda _j^{KED}(\mathbf{u} ) C_R(\mathbf{u} _i-\mathbf{u} _j) + \sum _{k=0}^4\mu _k^{KED}(\mathbf{u} ) x_k(\mathbf{u} _i)= C_R(\mathbf{u} _i-\mathbf{u} ) \quad i= 1, \ldots , 77 \end{aligned}$$

5 $$\begin{aligned} \sum _{j=1}^{77} \lambda _j^{KED}(\mathbf{u} )= 1 \\\sum _{j=1}^{77} \lambda _j^{KED}(\mathbf{u} ) x_1(\mathbf{u} _j)& = x_1(\mathbf{u} ) \\ \sum _{j=1}^{77} \lambda _j^{KED}(\mathbf{u} ) x_2(\mathbf{u} _j)& = x_2(\mathbf{u} ) \\ \sum _{j=1}^{77} \lambda _j^{KED}(\mathbf{u} ) x_3(\mathbf{u} _j)& = x_3(\mathbf{u} ) \\ \sum _{j=1}^{77} \lambda _j^{KED}(\mathbf{u} ) x_4(\mathbf{u} _j)& = x_4(\mathbf{u} ) \\ \end{aligned}$$

where the trend $m(\mathbf{u} )$ is modeled as a linear function of smoothly varying variables, the covariance function of separation vector between any pair of residuals estimated at SPA facility sites $C_R(\mathbf{u} _i-\mathbf{u} _j)$, and the covariance of a pair of facility in the survey and the prediction location $C_R(\mathbf{u} _i-\mathbf{u} )$. The four covariates $x_k, k=1, \ldots , 4$ denote the elevation, log-transformed population density, road density, and the distance to the nearest road, respectively.

*Statistical Calibration*: In both the local trend estimation and kriging models, some model predictions exceeded the range of general SR scores. Although it is possible that some dispensaries may have a lower or higher general SR score than measured values in the SPA facility survey, any model prediction outside the range of 0 and 100 is unacceptable. To avoid these spurious results and rectify a systematic bias if present, we performed a stochastic calibration using a subset of the census data, which hereafter referred to as testing sites. Note that the census data contain the locational information of complete health facilities in the study area, although their SR scores are unavailable except at SPA facility sites. However, the information on the facility type—Hospital, Health Center, Dispensary, and Clinic—was known at all health facilities registered in the census data. We created testing sites as a subset of the census data by applying a criterion based on the geographic proximity to the same type of facilities in SPA sample. Here, we assumed that two adjacent health facilities that were classified as the same type of health facility likely shared equivalent or similar general SR scores.

To operationalize this concept, we first examined each facility in the census if there was a SPA facility of the same type located within 5 km. If so, the facility in the census data would be included in the ‘testing sites’ and we assigned the general SR score of the nearest SPA facility to the testing site. Here we limited the search radius of the nearest same type SPA facility within 5 km based on a sensitivity analysis (see Appendix B for details). We examined the correlation between a pair of the same type of two nearest SPA health facilities’ SR scores with respect to a maximum search range, which are summarized in Table 4.

In the second step, we extracted predicted values at testing sites from a local trend model, SKLM, and KED models. Based on both the modeled and reference values at the testing sites, we developed a simple linear model to minimize the gap between model predictions and reference values. The fitted model was used to calibrate the model predictions across the study area.

*Performance Evaluation of Linkage Methods*: We evaluated the performance of the geostatistical linkage methods using the census of health facilities. Because general SR scores were available only at the subset of the census (77 facilities included in SPA), we could not directly use the census data in the evaluation. Instead, we used the information on the facility type as a proxy variable and quantified two evaluation metrics: (1) the prediction error at a subset of the census health facilities, i.e., testing sites identified in the calibration; (2) the classification error per facility type over the census of health facilities.

To calculate prediction error of the three algorithms of a generalized linear regression model, SKLM, and KED, we fitted the models with SPA data and obtained the predicted SR scores at testing sites. We also estimated general SR scores at testing sites using three commonly used linkage methods: administrative boundary link, Euclidean buffer link, and kernel density estimation methods. The details of the three linkage methods can be found in Skiles et al. [28], but we linked the two data sets within a district boundary (i.e., the administrative level 2 mapped in Fig. 1) for the administrative boundary link, and we created a 5 km buffer zone at each facility in testing sites for Euclidean buffer link. Lastly, the kernel density was created at a grid with cell size of 500 m using general SR scores of SPA data as a density variable. We calculated the absolute mean prediction error by taking the differences between linkage method specific predictions and reference values at testing sites and summarized them by calculating their mean.

For the classification error, we assessed if the predicted general SR score at each health facility is within the facility type specific range of general SR scores inferred from SPA. For each health facility in the census, we compared the general SR score predictions obtained from different linkage methods with the corresponding lower and upper bounds specific to the facility type. The comparison results were quantified as classification error across all the 563 health facility sites. If a model predicts a general SR score that is within the range of lower and upper bounds per facility type in Table 2, a value of 1 was assigned and 0, otherwise. We conducted this evaluation across all health facilities and computed the total success rate (0 to 100%) for each model.

### The effects of DHS cluster displacement on the linkage

Based on the proposed geostatistical linkage approach, we estimated the general SR at 40 DHS clusters. Under the consideration of the displacement each DHS cluster, we generated 100 sets of alternative and equally probable cluster locations. We simulated locations that are within 5 km for urban clusters and 10 km for rural clusters [2]. At each simulated DHS cluster, we estimated the general SR scores from the geostatistical methods and quantified their variability originating from the displacement. For the purpose of comparison, we also computed the general SR scores at the simulated cluster locations using other traditional methods and compared the absolute differences with the proposed geostatistical methods, as well as the variability.

All statistical analysis was conducted with R software (version 4.0.2) using *glm* for a trend estimation, *gstat* package for variogram modeling and spatial prediction, and *ks* package for kernel density estimation.

## Results

### Spatial distribution of health facilities

A total of 563 health facilities operated in the study region in 2019 that consist of 20 Hospitals (3.6%), 62 Health centers (11.0%), 10 Clinics (1.8%), and 471 Dispensaries (83.6%). In the SPA facility survey of 2014–2015, about 13.7% (*n* = 77) of these health facilities were selected, including 18 Hospitals (23.4%), 25 Health centers (32.5%), 2 Clinics (2.6%), and 32 Dispensaries (41.6%). As shown in Table 1, the difference between the SPA (sample) and census is substantial (77 versus 563 facilities) and the ratio of sample to census is substantially different per type of service they provide. For example, 18 hospitals were included in SPA survey out of a total of 20 hospitals in the study region (close to 90%), whereas only 32 out of a total of 471 Dispensaries (6.79%) are included in the SPA survey.

**Table 1:** The number of facilities of each type in SPA and Census

	Hospital	Health center	Clinic	Dispensary	Total
SPA	18	25	2	32	77
Census	20	62	10	471	563

The overall mean of the general SR of the SPA sampled facilities was 61.44 (57.71–65.17)^1^ with a standard deviation of 16.71. The range of SR scores varied by facility type as summarized in Table 2, although some of facility scores were similar to those of other types. It is clear that Hospitals had the highest scores (mean of 82.55 and SD of 7.23) and Dispensaries had the lowest scores (mean of 47.15 with SD 9.47) among the four facility types present in the study area. Specifically, the scores of Dispensaries were in the range of 29.15 and 68.83, whereas those of Hospital were in the range of 68.86 and 92.73. The lowest value of Hospital was higher than the maximum value of Dispensary. However, both the general SR of Clinics and that of Health centers overlapped with both Hospitals and Dispensaries. Overall, the scores of Health center (mean of 64.46) were similar to the mean (62.42) of general SR scores of Clinics.

**Table 2:** The range of general service readiness scores per health facility type

Facility Type	(Minimum, Maximum)	Mean	SD
Hospital	(68.86, 92.73)	82.55	7.23
Health center	(42.44, 80.27)	64.46	10.18
Clinic	(50.92, 73.91)	62.42	16.25
Dispensary	(29.15, 68.83)	47.15	9.47

*SD: Standard Deviation

A total of 98 facilities were included in the testing sites that have at least one SPA facility of the same type located within 5 km. The testing sites consist of 17 Hospitals, 29 Health centers, 2 Clinics, and 50 Dispensaries. Their reference values for general SR scores were in the range of 29.15 and 92.73 with the mean of 60.01 and standard deviation 16.15. By design, 77 facilities in the testing sites are from SPA.

### Geostatistical linkage

**Fig. 3:**
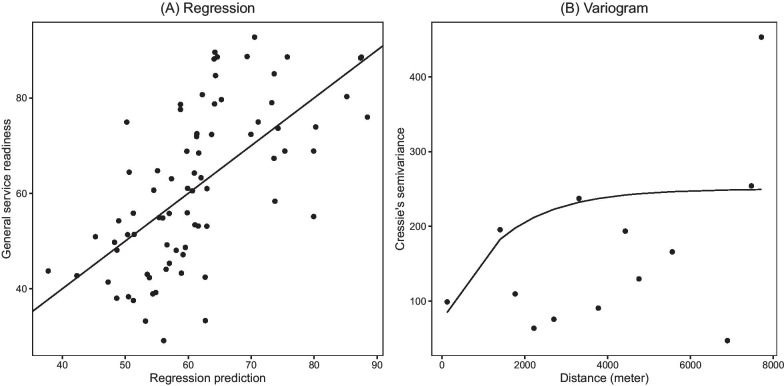
The regression for local trend (**A**) and semivariogram (**B**) of general service readiness (SR) with the model fitted

We developed a multivariate linear regression model for a local trend estimation and the regression results are summarized in Table 3. Both the population density and road density around the health facility have a positive and statistically significant association with the score, whereas both elevation and distance to major roads are negative and insignificant. The regression model fit is presented in Fig. 3A where the general SR scores are plotted with the corresponding local trend estimates.

**Table 3:** Regression results

	Estimate	Std. Error	*t* value	Pr(> |t|)
(Intercept)	63.94	6.88	9.29	0.00
Elevation	− 0.00	0.00	− 0.50	0.62
Log(pop.den)	4.35	1.10	3.95	0.00
Road.density	4.57	1.77	2.58	0.01
Distance to Roads (km)	− 0.34	0.51	− 0.67	0.50

The spatial correlations between general SR scores were modeled using the variogram of residuals obtained at the SPA sites. The resulting experimental variogram and its optimal model fit are presented in Fig. 3B, which are summarized by an exponential variogram model with a range 1428 m and the partial sill 180 with a nugget effect of 70.

**Fig. 4:**
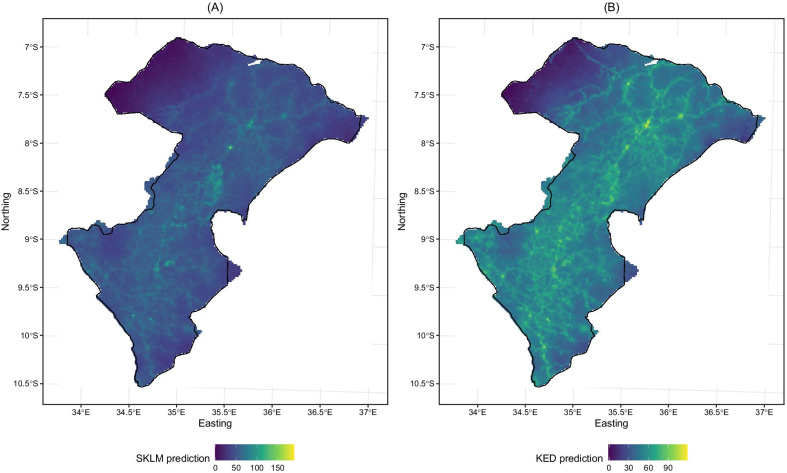
The surface of general service readiness scores obtained from (**A**) Simple Kriging with Varying Local Mean (SKLM) and (**B**) Kriging with External Drift (KED)

Based on the variogram model, we generated surfaces of predicted SR scores using two geostatistical prediction models. In addition, we created a surface of local trend that is solely based on a regression model as a baseline. The resulting prediction surfaces are shown in Fig. 4, where the lighter colors representing higher scores of the general SR were reproduced along the existing road networks and the villages with high population density. In comparison to SKLM in Fig. 4A, the KED predictions in Fig. 4B revealed the presence of local variations along the road networks. The SR scores in the top left corner of the study region are the lowest, where population is sparse as shown in Fig. 2C. It is also worth noting that some predicted scores from both SKLM and KED predictions are beyond the range of the original scores. To address this issue, we conducted the statistical calibration.

### Evaluation of geostatistical linkages

**Fig. 5:**
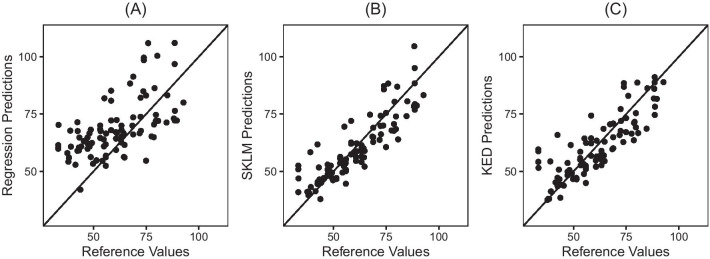
Prediction error from (**A**) Regression model, (**B**) Simple Kriging with Varying Local Mean (SKLM) and (**C**) Kriging with External Drift (KED)

Fig. 5 presents the scatter plots of the prediction versus reference values at testing sites. The results show that SKLM yield the smallest mean prediction error of 6.20, followed by KED (6.67). The local trend model estimated by a multivariate regression showed the poorest performance (10.40) among the three models. The mean prediction error of administrative boundary, buffer, and kernel density link was 11.91, 2.17, and 12.62, respectively, where the buffer link resulted in the minimum prediction error among all five methods.

In terms of classification error, SKLM yielded the highest success rate of 79% (classification error of 0.21), closely followed by the regression model (78%) and KED model of 77%. Among the three existing methods, their performance varies substantially with the administrative boundary model having a 69% of success rate, kernel density estimation 47 % and buffering method 31%, respectively.

### The effect of DHS cluster displacement on the linkage results

**Fig. 6:**
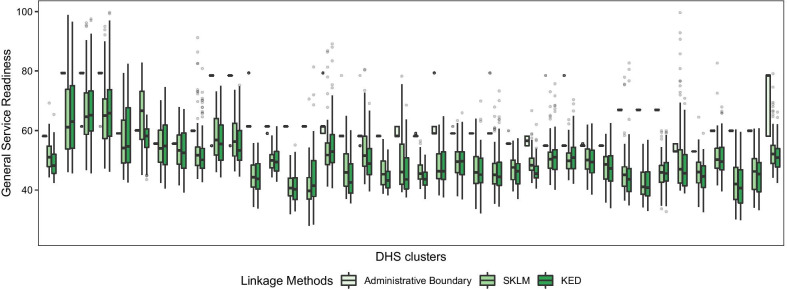
Linkage of SPA survey to DHS clusters under the consideration of their position displacement: Two geostatistical methods of SKLM and KED shaded in dark color are similar to each other, whereas the traditional method of Administrative boundary shows substantially different results

The effect of the cluster displacement on the estimation of general SR scores varies per DHS cluster, with the averages for SKLM and KED were 51.97 and 51.12, respectively. Meanwhile, the variability of these estimated scores, quantified by standard deviation, ranges from 2.99 to 13.44 for SKLM and 3.54 to 13.52 for KED. More substantial differences were found in comparison to the existing methods of administrative boundary, buffering, and KDE. We presented the variability of general SR scores at each DHS cluster obtained from two geostatistical methods (SKLM and KED) and administrative boundary method using a boxplot in Fig. 6. The estimates from two geostatistical linkage methods were invariant across all DHS clusters, although the variability of the estimated scores rising from the positional uncertainty varied per cluster. It is noticeable, meanwhile, that the administrative boundary linkage method was less sensitive to the displacement except for a few clusters, but yielded higher scores than geostatistical linkage methods.

## Discussion

We developed a geostatistical approach to link SPA and DHS datasets at a disaggregated level (i.e., the cluster-level), and presented the application of the proposed linkage methods using data from Tanzania. The proposed geostatistical linkage methods utilize the information on the spatial configuration of DHS clusters and SPA facilities similar to existing geospatial linkage methods, such as administrative boundary, buffering distance, and kernel density estimation, but our method is an improvement upon these. Specifically, the proposed linkage method explicitly accounts for the geographic, socio-economic, and demographic conditions of and around sampled health facilities in SPA as well as their spatial autocorrelations. They are critical to reducing the misclassification error introduced when SPA survey is linked to DHS clusters, given the SPA is typically conducted as a nationally representative sample of facilities except in a small number of countries where they have conducted a census of all facilities [32]. The knowledge gained from the spatial analysis of the SPA sample and its surrounding environments allows us to identify key determinants of existing health facility sites and to infer the locations of health facilities that are not sampled within SPA surveys. Consequently, it enables us to apply the proposed linkage approach to SPA surveys and DHS data at cluster levels while minimizing misclassification error.

In our investigation of the effect of geographic displacement of DHS cluster data on the linkage, we concluded that geographic displacement may lead to a biased link between the health service environment and population, as shown in the wide range of box lengths across DHS clusters of Fig. 6. However, the results also suggest that the effect of geographic displacement is relatively small in comparison to the uncertainty in the estimated service readiness due to the linkage methods used (i.e., geostatistical linkage method vs. administrative boundary method).

It is worth noting that proposed linkage methods are flexible and can be replicated with other service readiness or health indicators and in different settings. As key determinants for optimal health facility sites likely differ per region or country, it is necessary to modify the proposed multi-step geostatistical model to accommodate both the regional characteristics and the data availability. Many countries are at risk of not achieving the targets of the Sustainable Development Goal (SDG) 3 (to ensure healthy lives and promote wellbeing for all at all ages), because they continue to face challenges related to health services delivery and access. In order to improve service delivery and simultaneously, health outcomes and equity, a better understanding of how the health service delivery environment influences service utilization is needed. A first step in this involves linking health-sector and individual-level data for further analysis. As demonstrated using the data from two regions in Tanzania, our methods can contribute to better understanding of service utilization from an ecological perspective, but at a more local level, helping countries identify factors associated with the provision and utilization of services and address gaps to achieve SDG 3.

However, the proposed geostatistical approaches should be applied with caution when the SPA consists of small number of facilities. Empirical variograms constructed from small samples likely are unreliable and incapable of inferring underlying spatial structures of the process, which could consequently yield biased and inaccurate predictions. To avoid spurious results, we recommend to linking DHS data to SPA surveys at a disaggregated level only when a sufficient number of facilities are included in the SPA. Similarly, caution is required when interpreting the performance evaluation reported in the present study. Due to the absence of validation data, we created testing sites that include all SPA facilities and utilized their general SR scores in both model fitting and validation. Consequently, the linkage method based on buffering showed the best performance in the prediction error but the worst in classification error. This inconsistent finding suggested that the model performance evaluation was sensitive to the choice of validation data.

The current study has several strengths, including use of representative data; applicability of linkage methods to disaggregated levels unlike existing studies; use of rich data on population density, road networks, land use, and quality of services offered in health facilities; and potential for replication with other data nationally and in other settings. Nevertheless, the study has some limitations. First, we lack information on service readiness for facilities in the census. The SPA survey takes about 14% (77 sites out of 563) of the health facilities present in the study area. Potentially, the small sample size may have biased the inference of the spatial structure of the underlying process. Second, when the proposed approach is applied to a large study area, the stationarity assumption imposed on the relatively small study region has to be reevaluated. If the assumptions cannot be met, an alternative approach, including the division of study area to homogeneous regions, should be considered. Similarly, the spatial resolution of the prediction may affect the performance of the model. It should be also noted that the four covariates used for the present study are not exhaustive and may need revision when the proposed methods are applied to other regions. Finally, our SR information is based on general services. The readiness and availability of services may vary within a facility based on type of services being offered (e.g., family planning, vaccinations, delivery services). In this general exercise, we had limited our analysis to general SR to demonstrate the feasibility of our method.

In future research, the proposed linkage methods can be further improved by adopting sophisticated statistical methods and data mining techniques in combination with geostatistics. We also expect that a hybrid approach that involves both machine learning and geostatistics is applicable for different health service environments at regional and national levels. Lastly, we expect that the information on the access, utilization, and quality of health service estimated at DHS clusters will be used to assess the population health outcomes, including health status, health care-seeking behaviors, and policy questions about program impact and targeting related to population health.

## Conclusions

We developed a flexible approach for linking SPA and DHS data at a disaggregated level (i.e., the cluster-level), improving upon the limitations of existing linkage methods which are only applicable at regional scales. These linked data can be used to improve understanding of how the service delivery environment influences utilization of services, and subsequently health outcomes, at the individual-level for myriad health domains (child nutrition, vaccinations, fertility, sexual and reproductive health, etc.). This approach can be adapted with other health services indicators and in other settings. Thus, the contribution of the current study can help identify gaps in service provision to inform programming and policy in various settings. This improved knowledge can help countries improve health outcomes for their populations, improve equity, and achieve SDG 3.

## Data Availability

The datasets (SPA and DHS) analyzed during the current study are publicly available.

## Appendix A: Analysis of SPA data: general service readiness

For each facility, we used data from SPA to create a score summarizing service readiness comprising items related to amenities (power, improved water source, privacy, sanitation, communication, computer, emergency transportation), basic equipment (adult scale, child scale, thermometer, stethoscope, blood pressure apparatus, and light source); infection prevention (safe disposal of sharps, safe final disposal of infectious wastes, appropriate storage of sharps waste, appropriate storage of infectious waste, disinfectant, single use standard disposable or auto-disable syringes, soap and running water or alcohol based hand rub, latex gloves, guidelines for standard precautions); diagnostic capacity (hemoglobin, blood glucose, malaria, urine dipstick for protein, urine dipstick for glucose, HIV, syphilis rapid test, urine test for pregnancy); essential medicines (amlodipine tablet/calcium channel blocker, amoxicillin syrup/suspension or dispersible tablet, amoxicillin tablet, ampicillin power for injection, aspirin, beclomethasone, inhaler, beta blocker, arbamazepine tablet, ceftriaxone injection—second line injectable antibiotic, diazepam injection, enalapril tablet or ACE inhibitor, fluoxetine tablet, gentamicin injection, glibenclamide tablet, haloperidol tablet, insulin regular injection, magnesium sulfate injectable, metformin tablet, omeprazole tablet/pantoprazole, rabeprazole, oral rehydration solution, oxytocin injection, salbutamol inhalers, imvastatin tablet or other statin, thiazide, zinc sulfate tablets).

## Appendix B: Sensitivity analyses

We assessed the sensitivity of the assumption imposed by testing sites by examining a pair of SPA facilities of the same type with respect to their general SR scores and the separation distance. Depending the type of facility, a pair of facilities of the same type might be further apart and the physical distance may invalidate the assumption. Thus, we considered a maximum search distance and calculated the correlation coefficients between the general SR scores of selected pairs that are the same type and located within the maximum distance. If more than one facility of the same type is found within the search radius, we selected the nearest one. The results are summarized in Table 4 and it showed that as a maximum search distance increases, the correlation between the general SR scores of the pairs decreases and the total number of pairs selected increases. We have chosen 5000 m in the present study under the consideration of both the number of pairs of the facilities (the size of testing sites) and the correlation coefficients.

**Table 4:** Sensitivity analysis: correlation coefficients of general SR scores

Max. Distance (m)	No. pairs of the same type facilities	Correlation coefficient
1000	79	0.99
2000	88	0.84
3000	91	0.83
4000	95	0.81
5000	95	0.81
6000	95	0.81
7000	97	0.81
8000	107	0.68
9000	112	0.65
10000	115	0.65

## Abbreviations

SPA: Service Provision Assessment
DHS: Demographic and Health Survey
SR: service readiness
LMICs: low- and middle-income countries
SKLM: simple kriging with varying local mean
KED: kriging with an external drift

## Footnotes

1 95% confidence intervals.
